# The Pharmacological Effect of Hemin in Inflammatory-Related Diseases: Protocol for a Systematic Review

**DOI:** 10.2196/48368

**Published:** 2023-11-16

**Authors:** João Estarreja, Gonçalo Caldeira, Inês Silva, Priscila Mendes, Vanessa Mateus

**Affiliations:** 1 H&TRC–Health and Technology Research Center ESTeSL–Escola Superior de Tecnologia da Saúde de Lisboa Instituto Politécnico de Lisboa 1990-096 Lisbon Portugal; 2 iMed.ULisboa Faculdade de Farmácia Universidade de Lisboa Lisbon Portugal

**Keywords:** hemin, inflammation, animal models, nonclinical studies, in vivo

## Abstract

**Background:**

Hemin is a commonly used drug in the treatment of acute attacks of porphyria, due to its capability of restoring normal levels of hemoproteins and respiratory pigments. In addition, this drug has demonstrated the capacity to induce the heme oxygenase (HO) enzyme. At the moment, there are 3 known HO isoenzymes in mammals: HO-1, HO-2, and HO-3. The first of these shows cytoprotective, antioxidant, and anti-inflammatory effects. Currently, medicines used in inflammatory disorders have increased toxicity, especially over longer time frames, which highlights the need to investigate new, safer options. Indeed, the current nonclinical evidence demonstrates the potential that hemin has a significant anti-inflammatory effect in several animal models of inflammation-related diseases, such as experimental colitis, without significant side effects. However, the underlying mechanism(s) are still not fully understood. In addition, past nonclinical studies have applied different therapeutic regimens, making it relatively difficult to understand which is optimal. According to the literature, there is a lack of review articles discussing this topic, highlighting the need for a summary and analysis of the available preclinical evidence to elucidate the abovementioned issues. Therefore, a qualitative synthesis of the current evidence is essential for the research and medical communities.

**Objective:**

This systematic review aims to summarize and analyze currently available nonclinical data to ascertain the potential anti-inflammatory effect of hemin in animal models.

**Methods:**

Throughout the development of this protocol, we followed the Preferred Reporting Items for Systematic Reviews and Meta-Analyses (PRISMA) protocol. The comprehensive search strategy will be carried out in MEDLINE (PubMed), Web of Science, and Scopus without any filters associated with publication date. Only in vivo, nonclinical studies that evaluated the potential anti-inflammatory effect of hemin will be included. The evaluated outcomes will be the observed clinical signs, inflammatory and other biochemical markers, and macroscopic and microscopic evaluations. To analyze the potential risk of bias, we will use the risk of bias tool developed by the Systematic Review Centre for Laboratory Animal Experimentation (SYRCLE).

**Results:**

Currently, it is not possible to disclose any results since the project is still in initial steps. More specifically, we are currently engaged in the identification of eligible articles through the application of the inclusion and exclusion criteria. The work was initiated in April 2023, and it is expected to be finished at the end of 2023.

**Conclusions:**

Concerning the major gap in the literature regarding the underlying mechanism(s) and treatment-related properties, this systematic review will be essential to clearly summarize and critically analyze the nonclinical data available, promoting a clearer vision of the potential anti-inflammatory effect of hemin.

**Trial Registration:**

PROSPERO CRD42023406160; https://www.crd.york.ac.uk/prospero/display_record.php?RecordID=406160

**International Registered Report Identifier (IRRID):**

PRR1-10.2196/48368

## Introduction

Hemin, or ferriprotoporphyrin IX chloride, is an iron-containing metalloporphyrin that has been commonly used in clinical practice for several decades in the treatment of acute attacks of hepatic porphyria [1,2]. The development of porphyria is closely related to deficiency in the heme biosynthesis pathway, resulting in a lack of heme, which is necessary to produce several hemoproteins. In addition, heme precursors, which are directly and indirectly toxic to the human body, can also accumulate [3,4]. Treatment with hemin shows the capability of reducing heme deficiency by suppressing delta-aminolaevulinic acid synthase activity. Furthermore, this treatment also promotes the reduction of porphyrins and toxic precursors of heme [4,5]. Once hemin restores the normal levels of hemoproteins and respiratory pigments, the biological disturbances observed in patients with porphyria are attenuated [4,5].

However, the administration of hemin can also induce the expression of the heme oxygenase (HO) enzyme, which is known as a rate-limiting enzyme for heme catabolism and is responsible for the production of biliverdin, free iron, and carbon monoxide [2,6,7]. Currently, there are 3 identified HO isoenzymes in mammals: HO-1, HO-2, and HO-3 [2,6]. HO-1 is expressed as a response to tissue damage and demonstrates cytoprotective action capable of inhibiting the inflammatory response and oxidative stress [8]. On the other hand, the HO-2 and HO-3 isoenzymes are expressed constitutively in tissues involved in heme catabolism, which regulates the normal functioning of cells [7,9]. Considering the nonclinical evidence currently available, it has been suggested that HO-1 concentration and HO-1 mRNA are significantly elevated in cases of inflammation, such as in active ulcerative colitis [2,6,10]. In fact, through histological studies, it has been observed that HO-1 expression occurs mainly in macrophages; however, it can also be found in epithelial cells when there is an inflammatory response [11,12]. The capability of hemin to induce the expression of HO-1 makes it an interesting drug to evaluate for use in inflammatory-related diseases to ascertain its anti-inflammatory effect.

Considering the high toxicity of commonly used drugs for inflammation-related diseases, especially over the longer term, it is essential to investigate safer approaches that have comparable efficacy [13]. Several nonclinical studies have demonstrated that hemin is a significant anti-inflammatory without relevant side effects, highlighting it as a new potential pharmacological approach for the future. Considering the vast number of therapeutic regimens adopted in nonclinical studies, different animal models of disease, and different evaluated outcomes, it is essential to perform a review to clearly analyze and criticize the current evidence. Thus, this project will aim to develop a systematic review of the potential anti-inflammatory effect of hemin by summarizing and analyzing the nonclinical data currently available.

## Methods

### Overview

This protocol follows the Preferred Reporting Items for Systematic Reviews and Meta-Analyses (PRISMA) guideline [14]. During the development of the systematic review, 2 authors from the research group will be responsible for all steps concerning the selection of studies, including the title and abstract screening and assessment of study eligibility, as well as the data extraction process. In case of discrepancies during these steps, a third reviewer will be included to make a final decision. The project is currently ongoing, having been initiated in April 2023 with an anticipated completion date at the end of 2023.

### Eligibility Criteria

Studies will be included for further analysis if they meet the follow PICOD (population, intervention, comparator, outcomes, study design) eligibility criteria:

- Population: Studies that use rodent models of inflammatory-related diseases. There will be no limitations in terms of species, strain, age, sex, or body weight since the purpose of this study is to analyze and synthesize all nonclinical data available.
- Intervention: Studies that administer hemin at any dosage, frequency, or treatment duration and with any route of administration in animal models of inflammatory-related diseases will be included, while studies that combine hemin treatment with another active drug or drugs will be excluded to clearly evaluate hemin’s anti-inflammatory effect, excluding potential bias related to the use of other medicine.
- Comparator: Studies will be included if they have a nonexposed control group that is treated with any other pharmacological molecule, or even a placebo or usual care.
- Outcomes: All biochemical markers related to inflammation, with and without hemin treatment, will be analyzed. Studies with no relevant reported outcomes or that evaluate hemin administration for diseases as part of a summary of product characteristics will not be included.
- Study design: Only analytic, experimental, in vivo, nonclinical studies will be included.

Furthermore, studies written in languages other than English will not be included.

### Information Sources and Search Strategy

The biomedical electronic databases used for the highly sensitive search strategy will be MEDLINE (PubMed), Web of Science, and Scopus. The search will not be limited in terms of publication date. A comprehensive search strategy will be developed using descriptors related to 3 key terms: *hemin*, *inflammation*, and *nonclinical study*, as well as their synonyms, combined with the Boolean operators “AND” and “OR” to identify and select the eligible studies. The search strategies adopted for each biomedical electronic database are available in Multimedia Appendix 1.

### Study Selection

Upon the application of the comprehensive search strategy in each database, the retrieved articles will be exported from the MEDLINE (PubMed), Web of Science, and Scopus databases to a systematic reviews web application (Rayyan QCRI; Rayyan). The first step will be to detect and exclude duplicates, and afterward the titles and abstracts will be analyzed by 2 independent reviewers in order to select relevant and potentially eligible studies according to the inclusion and exclusion criteria. After this process is completed, the same 2 independent reviewers will assess the full text of each study, deciding whether the article is eligible or not considering the inclusion and exclusion criteria. In case of discrepancies and lack of consensus between the 2 reviewers in these steps, a third reviewer will be included to make a final decision. The process of selection will be summarized using a PRISMA flowchart.

### Data Collection Process

Upon selection of eligible studies, the 2 independent reviewers will extract them to a standardized data extraction document in Excel (Microsoft) that will be developed to extract the data of interest, including article identification (authors’ names and year of publication), animal-related parameters, disease-related parameters, hemin treatment conditions, and outcomes of interest. Once again, in case of discrepancies between the reviewers, a third reviewer will be included to make the final decision. The data of interest will be extracted from the text, graphs, and tables from each included article.

### Data Items

#### Population

We will only consider studies based on animal models, and the data of interest to be extracted will be related to species, strain, sex, age, and body weight.

#### Intervention

We will only consider studies that administer hemin to the subjects. The parameters of interest will be dosage, frequency of administration, route of administration, and duration of treatment.

#### Comparator

We will not consider any comparator-related parameters.

#### Outcomes

The data concerning the outcomes will be related to observed clinical signs (dichotomous measures), inflammatory and biochemical markers (continuous quantitative measures), macroscopic evaluation (dichotomous measures), and microscopic evaluation (dichotomous measures).

### Study Design

Throughout the analysis of the in vivo nonclinical studies, the data of interest to be extracted will be related to identification of inflammatory-related diseases, their severity, and their chronicity.

### Risk of Bias Assessment

The potential risk of bias will be identified and analyzed using the risk of bias tool of the Systematic Review Centre for Laboratory Animal Experimentation (SYRCLE) [15]. For each study, each component of the SYRCLE tool and the global study rating will be graded as low, moderate, or high. The final grade will be also decided considering an average of all individual components. The reporting quality and the risk of bias assessments will be independently performed by 2 different reviewers, after the data collection process is completed. Any disagreements will be discussed and arbitrated by a third reviewer.

### Registration and Reporting

This systematic review protocol has been registered in an international prospective register of systematic reviews (PROSPERO CRD42023406160). Throughout the development of this systematic review, any potential modification of the protocol will be reported.

## Results

Currently, the project has included 50 of the 1110 retrieved articles for qualitative synthesis based on the established inclusion and exclusion criteria. The data of interest are being carefully extracted by the 2 independent reviewers. The work is being performed by a pharmacologist team without any external funding and was initiated in April 2023. It is expected to be finished at the beginning of 2024.

## Discussion

Current systematic reviews make it is possible only to determine that there is substantial variability in protocols for treatment with hemin, such as the dose, frequency of administration, and duration. However, even with different therapeutic regimens, this drug has shown a significant anti-inflammatory effect, mainly related to its capability to induce HO-1 without any relevant side effects.

It is clear that the majority of pharmacological approaches used in chronic inflammatory-related diseases aim to induce and maintain a remission phase in patients [13]. However, these approaches normally have several side effects, especially considering the long time frame of the treatment regimens, which can significantly decrease the overall quality of life of patients [13]. Thus, it is essential to investigate safer pharmacological tools, either by developing new drugs or by repurposing already accepted and extensively studied ones. Hemin is one of the latter, and it represents an interesting pharmacological approach to treatment of inflammatory-related diseases, considering its efficacy and safety over the short or long term. Indeed, several nonclinical studies have already demonstrated that hemin has a significant anti-inflammatory effect in different contexts, such as in inflammatory bowel disease [2,6,16,17], kidney dysfunction [18-20], sepsis [21-23], arthritis [24,25], pancreatitis [26-28], cardiac infarction [29], and airway inflammation [30-32].

We emphasize a major limitation regarding the methodology of this project, which is the absence of clinical evidence. Indeed, it would be interesting to analyze clinical data regarding the potential anti-inflammatory effect of hemin; however, the number of studies is relatively small and there have been no clinical trials on this topic. Therefore, we only considered nonclinical in vivo studies, since this approach allowed for the most controlled environment possible.

In conclusion, the main findings from this project may have a pivotal role in the research and medical communities, since we will summarize and discuss, for the first time, preclinical evidence regarding the potential anti-inflammatory effect of hemin. Indeed, considering the need to investigate safer pharmacological approaches for inflammatory diseases, this review should highlight an interesting new tool for future consideration, revealing its efficacy and safety as demonstrated in animal models.

## Appendix

Multimedia Appendix 1 Search strategies for each biomedical electronic database.

## Abbreviations

HO: heme-oxygenase
PICOD: population, intervention, comparator, outcomes,
PRISMA: Preferred Reporting Items for Systematic Reviews and Meta-Analyses.
SYRCLE: Systematic Review Centre for Laboratory Animal Experimentation

## Data Availability

All data generated or analyzed during this study are included in this published article and its supplementary information files.
