# Supplementary material for: The Pharmacological Effect of Hemin in Inflammatory-Related Diseases: Protocol for a Systematic Review
Source: JMIR Res Protoc. 2023 Nov 16;12:e48368. doi: 10.2196/48368 (PMC10690530; doi:10.2196/48368)
Supplement: Multimedia Appendix 1 [file resprot_v12i1e48368_app1.pdf]

Supplementary Material

Comprehensive search expression applied in the biomedical electronic bases MEDLINE (Pubmed), Web of Science and Scopus, including the results obtained:

MEDLINE (PubMed) - Research expression:

(((hemin[MeSH Terms]) OR (hemin[Title/Abstract])) AND ((inflammation[MeSH Terms]) OR (inflammat*[Title/Abstract])) AND ((disease models, animal[MeSH Terms]) OR ("animal model*") OR ("disease model*") OR ("animal experimentation*") OR ("nonclinical stud*") OR ("non-clinical stud*") OR ("preclinical stud*") OR ("pre-clinical stud*") OR ("in vivo") OR (rat*[Title/Abstract]) OR (mouse[Title/Abstract]) OR (mice[Title/Abstract]) OR (rodent*[Title/Abstract])))

Pubmed - Results: 272 results at March 7th 2023

Web of Science - Research expression:

(TI=(hemin)) OR (AB=(hemin)) AND (TI=(inflammat*)) OR (AB=(inflammat*)) AND (ALL=("animal model*")) OR (ALL=("disease model*")) OR (ALL=("animal experimentation*")) OR (ALL=("nonclinical stud*")) OR (ALL=("non-clinical stud*")) OR (ALL=("preclinical stud*")) OR (ALL=("pre-clinical stud*")) OR (ALL=("in vivo")) OR (TI=(rat*)) OR (AB=(rat*)) OR (TI=(mouse)) OR (AB=(mouse)) OR (TI=(mice)) OR (AB=(mice)) OR (TI=(rodent*)) OR (AB=(rodent*))

Web of science - Results: 283 results at March 7th 2023

Scopus - Research expression:

TITLE-ABS-KEY (hemin) AND TITLE-ABS-KEY (inflammat*) AND ALL ("animal model*") OR ALL ("disease model*") OR ALL ("animal experimentation*") OR ALL ("nonclinical stud*") OR ALL ("non-clinical stud*") OR ALL ("preclinical stud*") OR ALL ("pre-clinical stud*") OR ALL ("in vivo") OR TITLE-ABS-KEY (rat*) OR TITLE-ABS-KEY (mouse) OR TITLE-ABS-KEY (mice) OR TITLE-ABS-KEY (rodent*)

Scopus - Results: 555 results at March 7th 2023
